# Point-of-care ultrasonography in Brazilian intensive care units: a national survey

**DOI:** 10.1186/s13613-018-0397-3

**Published:** 2018-04-20

**Authors:** José Augusto Santos Pellegrini, Ricardo Luiz Cordioli, Ana Cristina Burigo Grumann, Patrícia Klarmann Ziegelmann, Leandro Utino Taniguchi

**Affiliations:** 1 Department of Critical Care Medicine, Hospital de Clínicas de Porto Alegre, Universidade Federal do Rio Grande do Sul, Porto Alegre, Brazil; 2 Department of Critical Care Medicine, Hospital Israelita Albert Einstein, São Paulo, Brazil; 3 Department of Critical Care Medicine, Alemão Oswaldo Cruz Hospital, São Paulo, Brazil; 4 Department of Critical Care Medicine, Nereu Ramos Hospital, Florianópolis, Brazil; 5 Statistics Department and Post-Graduation Program in Epidemiology, Universidade Federal do Rio Grande do Sul, Porto Alegre, Brazil; 6 Department of Critical Care Medicine, Hospital das Clínicas de São Paulo, FMUSP, São Paulo, Brazil; 7 Department of Intensive Care Unit, Hospital Israelita Albert Einstein, 627, Albert Einstein St., São Paulo, 05652-900 Brazil

**Keywords:** Ultrasonography, Critical care, Survey

## Abstract

**Background:**

Point-of-care ultrasonography (POCUS) has recently become a useful tool that intensivists are incorporating into clinical practice. However, the incorporation of ultrasonography in critical care in developing countries is not straightforward.

**Methods:**

Our objective was to investigate current practice and education regarding POCUS among Brazilian intensivists. A national survey was administered to Brazilian intensivists using an electronic questionnaire. Questions were selected by the Delphi method and assessed topics included organizational issues, POCUS technique and training patterns, machine availability, and main applications of POCUS in daily practice.

**Results:**

Of 1533 intensivists who received the questionnaire, 322 responded from all of Brazil’s regions. Two hundred and five (63.8%) reported having access to an ultrasound machine dedicated to the intensive care unit (ICU); however, this was more likely in university hospitals than in non-university hospitals (80.6 vs. 59.6%; risk ratio [RR] = 1.35 [1.16–1.58], *p* = 0.002). The main applications of POCUS were ultrasound-guided central vein catheterization (49.4%) and bedside echocardiographic assessment (33.9%). Two hundred and fifty-eight (80.0%) reported having at least one POCUS-trained intensivist in their staff (trained units). Trained units were more likely to perform routine ultrasound-guided jugular vein catheterization than non-trained units (38.6 vs. 16.4%; RR = 2.35 [1.31–4.23], *p* = 0.001). The proportion of POCUS-trained intensivists and availability of a dedicated ultrasound machine were both independently associated with performing ultrasound-guided jugular vein catheterization (RR = 1.91 [1.32–2.77], *p* = 0.001) and (RR = 2.20 [1.26–3.29], *p* = 0.005), respectively.

**Conclusions:**

A significant proportion of Brazilian ICUs had at least one intensivist with POCUS capability in their staff. Although ultrasound-guided central vein catheterization constitutes the main application of POCUS, adherence to guideline recommendations is still suboptimal.

**Electronic supplementary material:**

The online version of this article (10.1186/s13613-018-0397-3) contains supplementary material, which is available to authorized users.

## Background

Point-of-care ultrasonography (POCUS) has recently become a useful and widely disseminated tool that intensivists are incorporating into clinical practice. Assimilation timeframe varies according to the geographic, economic, and structural characteristics in which the practitioner is working. POCUS assists physicians to diagnose different causes of clinical deterioration and respiratory and/or hemodynamic failure, to tailor medical interventions (e.g., fluid therapy and mechanical ventilation adjustments), and to guide invasive procedures [1–3].

Despite guidelines that recommend the use of POCUS in different scenarios of critical care [3, 4], the incorporation of ultrasonography into clinical practice is not straightforward. For central venous catheterization (CVC), recent data showed that 18% of French intensivists reported that they routinely use ultrasonography [5], while 44% and 37% of emergency medicine specialists [6] and anesthesiologists [7], respectively, reported never using ultrasonography for CVC guidance.

Previous studies assessed the implementation of POCUS in critical care [5, 8–10]. Methodologies for collecting data were varied: while some authors applied electronic mailing and waited for spontaneous return, or based their findings on self-reported previous experience, others adopted cross-sectional epidemiological sampling consisting of punctual observations [9]. Similar studies revealing ultrasonographic patterns of use in intensive care units (ICUs) in developing regions are still lacking.

Brazil’s large territory and policies of public health system organization lead to challenges in access of health care personnel to the population, access to medical education, and the incorporation of new technologies into clinical practice. Therefore, precise information about regionalization, training methods, and preferential applications could help guide national entities in achieving more efficient dissemination of ultrasonography across Brazilian ICUs, potentially improving the quality of delivered care.

Surveys are standard tools that are increasingly used for assessing various aspects of health care, including educational, technological, and organizational aspects [11], as well as for investigating translation from scientific research to clinical practice [12]. Consisting of descriptive or explanatory questions, surveys can support the incorporation of medical evidence in current patient care.

Therefore, the purpose of our study was to utilize a survey to assess current practice and education of POCUS by Brazilian intensivists, as well as to assess the dissemination of ultrasonography and main applications in ICUs across Brazil. By evaluating the frequency of use and barriers of implementing ultrasonography, we can identify gaps in medical education and incorporate recommended clinical practices to critical care.

## Methods

This study was conducted with the logistic support of AMIBNet (the Brazilian network of research in intensive care) and *Ecografia em Terapia Intensiva* (ECOTIN), the national training program of POCUS for intensivists. No financial support was received from any source.

The ECOTIN program is an initiative of the Brazilian Intensivists Medical Association (AMIB), which was conceived in 2010. It consists of a board of POCUS experts who developed a teaching method and conduct short duration courses throughout Brazil. The ECOTIN course encompasses physical principles of ultrasound, knobology, echocardiographic and lung ultrasound basic techniques, as well as incorporating supervised, practical activities to allow participants to demonstrate expertise acquisition.

Questions were selected using the Delphi method. Four of the authors developed a set of questions of interest, which were then subjected to three rounds of appreciation. One author served as a facilitator, assessing agreement among the other three authors and providing feedback between rounds. Rounds were stopped when consensus was reached for all questions in the set. No physical meetings occurred. All of the panel members are formally certified intensivists and well-known experts in POCUS techniques and teaching. Three of the panel members are from the board of the ECOTIN group.

The survey consisted of 32 questions (Additional file 1) assessing the geographic location, type of hospital and type of ICU, availability of an ultrasound (US) machine, training in POCUS techniques, use and daily practice of US-guided CVC and other applications of POCUS (e.g., echocardiography, measuring the optic nerve sheath diameter, lung and abdominal studies), medical residents’ education in POCUS, and perceived barriers to the implementation of ultrasonography. All questions focused on POCUS performed by intensivists, not on complementary exams done by other physicians (e.g., radiologist or cardiologist). Skip logic was used when appropriate to ease the burden on respondents.

A web-based platform (SurveyMonkey^®^, www.surveymonkey.com) was used for the survey according to recent recommendations [12]. Initially, a group of 12 ICU physicians tested the questionnaire. After an interval of 3 weeks, a retest was performed by the same 12 physicians to verify reliability. The survey was physician-centered and was directly sent to intensivists subscribed to the AMIBnet mailing list. The questionnaire was available for 6 months (from September 2016 to March 2017). Reminders were sent via e-mail to potential participants on three occasions, every 2 months.

Survey respondents were stratified according to the training status of POCUS: trained versus non-trained. Trained status was dependent on having at least one intensivist with formal POCUS training working in the ICU staff. Additionally, questions gathered data on the proportion of staff that were trained in POCUS.

### Statistical analysis

Categorical variables were presented as absolute numbers and percentages and compared using the Chi-square test with standardized adjusted residuals analysis (for tables larger than 2 × 2) and the Fisher exact test (for 2 × 2 comparisons). A two-sided *p* value < 0.05 was considered statistically significant. Risk ratios (RRs) and 95% confidence intervals were calculated for associated measurements.

Multivariate analysis through Poisson linear models with robust estimation were constructed to identify variables that are independently associated with an important quality-of-care marker: US-guided internal jugular vein (IJV) puncture, in compliance with international guidelines [13–15]. A priori interest factors were those plausibly associated: the type of institution (university vs. non-university), presence of an intensivist on a daily basis, availability of a dedicated US machine, intensivists’ formal certification in critical care, proportion of POCUS-trained intensivists (low vs. high level), and payoff. For the construction of the multivariate model, we used forced simultaneous entry—all candidate variables remained in the model regardless of statistical significance. Outputs from this analysis are summarized as RRs.

All analyses were performed using Statistical Package for Social Science (SPSS), version 21.0 (IBM Corp., Armonk, NY, USA).

## Results

### Characteristics of the study population

From September 2016 to March 2017, 1533 intensivists were contacted by electronic mail. Of these, 322 responded (20.7% response rate) from all Brazilian regions. Of the units where respondents were working, private hospitals represented 46% while clinical-surgical units represented 72%. Three hundred and three units (94%) had a certified ICU physician attending daily rounds (Table 1).

**Table 1:** Baseline characteristics of the study population

Characteristic	*n* (322)
Region within Brazil	
Southwest	191 (59.7)
South	58 (18.1)
Northeast	43 (13.4)
Central-West	19 (5.9)
North	9 (2.8)
Hospital’s type	
Private	148 (46)
Public	107 (33.2)
University	67 (20.8)
ICU type	
Mixed, clinico-surgical	231 (72)
Clinical	43 (13.4)
Pediatrics	22 (6.8)
Surgical	13 (4.0)
Trauma	12 (3.7)
Number of beds	
< 10	130 (40.5)
11–20	107 (33.2)
21–40	47 (14.6)
> 41	37 (11.5)
Attendance of a certified intensivist during daily rounds	
Full-time (morning and afternoon)	151 (46.9)
Part time (morning or afternoon)	152 (47.2)
None	19 (5.9)

*ICU* intensive care unit

Two hundred and five (63.8%) of the respondents stated that they had access to a US machine dedicated to the ICU (Table 2). There were disparities throughout Brazil’s territory in this subject (*p* = 0.017). Availability of a dedicated US machine was more likely in university than in non-university hospitals (80.6 vs. 59.6%, respectively; RR = 1.35 [1.16–1.58], *p* = 0.002).

**Table 2:** POCUS characteristics

Characteristic	*n* (322)
Dedicated ultrasound machine availability	
No	116 (36.1)
Yes, without Doppler imaging	35 (10.9)
Yes, with Continuous and Pulsatile Doppler	63 (19.6)
Yes, with Continuous, Pulsatile and Tissue Doppler	107 (33.3)
Proportion of patients assessed by POCUS on a regular basis	
< 10%	124 (39)
11–25%	100 (31.4)
26–50%	55 (17.3)
51–75%	22 (6.9)
> 75%	17 (5.3)
POCUS type	
US-guided CVC insertion	156 (49.4)
Cardiac assessment	107 (33.9)
Lung	27 (8.5)
Abdominal	26 (8.2)
POCUS recording	
No recording	170 (54.5)
Registered on patient’s records	102 (32.7)
Electronically stored and registered on patient’s records	47 (14.6)
Medical report formally provided	16 (5.1)
Payoffs	
None	278 (90.3)
Only for the institution	13 (4.2)
Only for the physician	4 (1.3)
For both the institution and the physician	13 (4.2)

*POCUS* point-of-care ultrasound, *US* ultrasound, *CVC* central vein catheterization

US-guided CVC was the main indication of POCUS, representing 49.4% of indications, followed by bedside echocardiography (33.9%). Pleuropulmonary and abdominal ultrasonographic examinations were infrequently reported (8.5 and 8.2%, respectively). According to 59% of the respondents, chest x-rays are performed on a daily basis in more than 50% of the patients. This was negatively associated with the frequency of lung ultrasound examination (*p *=0.028).

Sixteen respondents (5.1%) stated that they use a prespecified form for summarizing data concerning examinations. The exam results images and clips) were electronically recorded 14.6% of the time.

### Competence and training of POCUS

Two hundred fifty-eight (80.0%) respondents reported to have at least one intensivist with formal POCUS training working in their staff (Table 3). We designated these as trained units for the following comparisons. We did not identify significant differences across Brazil’s territory according to training. The most frequently reported training structure was one- to two-day courses (65%).

**Table 3:** Medical trainment in POCUS

Characteristic	*n* (322)
Trainment status	
Yes—ECOTIN	60 (18.7)
Yes—ECOTIN and other courses	111 (34.6)
Yes—only other courses	87 (27.1)
None	63 (19.6)
Trainment modality	
1–2 days courses	163 (65.2)
2 days–1 week	46 (18.4)
1 week–1 month	8 (3.2)
> 1 month	33 (13.2)
Proportion of medical staff trained in POCUS	
< 10%	125 (50)
11–25%	68 (27.2)
26–50%	38 (15.2)
51–75%	11 (4.4)
> 76%	8 (3.2)
Medical residents	
Yes—Critical care medicine	138 (44.1)
Yes—Other specialties	76 (24.3)
None	99 (31.6)
Medical residents POCUS training modality	
Practice-based only	118 (54.1)
Lecture-based trainment available	44 (20.2)
Simulation-based	26 (11.9)
No trainment	30 (13.8)

*POCUS* point-of-care ultrasound, *ECOTIN* ecografia em terapia intensiva

ECOTIN trained 53% of the participants. The implementation of the ECOTIN training method was associated with the type of hospital: 67 and 49% of university and non-university hospital workers reported ECOTIN training, respectively (RR = 1.35 [1.1–1.67], *p* = 0.013). ECOTIN training prevalence was heterogeneous among Brazil’s regions (*p* = 0.001).

Medical residents were present in 69% of the units, including critical care medicine residents in 44%. There were no structured training modules for US-guided CVC in 54% of cases. Twenty-six respondents (8.1%) said that their residents use simulation techniques to learn how to perform US-guided CVC.

As aforementioned, we stratified respondents and their respective units into trained versus non-trained units based on self-reporting of training. We observed that the training status in POCUS affected the pattern of some answers (Table 4), such as availability of a dedicated US machine, proportion of patients assessed by POCUS and the density of certified intensivists.

**Table 4:** Competence and POCUS

Characteristic	Trained (258)	Not trained (63)	Risk Ratio (95% CI)	*p* value
Dedicated ultrasound machine	184 (71.3)	22 (34.9)	2.04 (1.44–2.89)	< 0.001
High intensity of certified intensivists	160 (62)	21 (33.3)	1.86 (1.29–2.67)	< 0.001
> 10% of patients assessed by POCUS on daily basis	165 (64.7)	29 (46.8)	1.38 (1.04–1.83)	0.013
Routine US-guided IJV catheterization	96 (38.6)	10 (16.4)	2.35 (1.31–4.23)	0.001
Payoff	14 (5.6)	16 (27.6)	0.20 (0.11–0.39)	< 0.001

*POCUS* point-of-care ultrasound, *CI* confidence interval, *US* ultrasound, *IJV* internal jugular vein

**Fig. 1:**
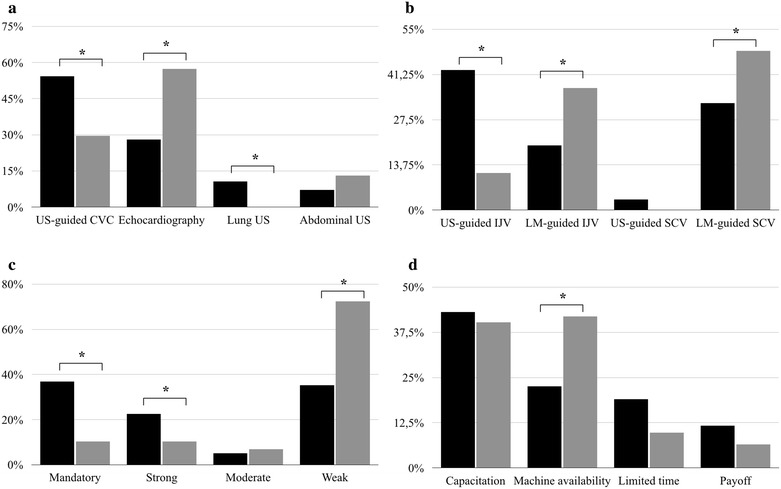
Black bars represent trained units; gray bars represent non-trained units. **a** main types of POCUS application; **b** preferential venous access site; **c** perceived strength of recommendation for US-guided jugular vein catheterization; and **d** main barriers for ultrasound dissemination. See text for full explanation. *differences in prevalences with *p *< 0.05. *US* ultrasound, *CVC* central vein catheterization, *IJV* internal jugular vein, *SCV* subclavian vein, *LM* landmark

The most frequent POCUS applications were distinct according to individual capability (*p* < 0.001; Fig. 1a): US-guided CVC and pulmonary ultrasonography was positively associated with trained individuals.

The status of POCUS training was negatively associated with exam payoff: fourteen (5.6%) of the trained units reported payoffs, compared to 27.6% of the non-trained units (RR = 0.20 [0.11–0.39], *p* < 0.001).

### US-guided CVC

According to the study participants, 58.5% of the units dealt with a monthly incidence density of less than 200 catheter-days and, in 54.5% of the cases, a catheter-related bloodstream infection of less than 2 events per 1000 catheter-days. The two most common approaches for CVC were the US-guided IJV (36.2%) and landmark-guided subclavian vein approaches (35.6%).

Respondents were questioned about the frequency with which CVC is US-guided in their units. In 22.8% of cases, IJV catheterization was “always” guided (in line with international guidelines). The most frequent answer was “only in specific situations” (45%). One hundred thirty-one (40.7%) of the participants classified the recommendations for routine use of ultrasonography when catheterizing the IJV as “weak”. For subclavian access, 78% of the respondents reported “rarely” using US, while 5% reported routinely using it; two hundred twenty-seven (70.5%) physicians classified the recommendation supporting US for subclavian puncture as “weak”.

Patterns of preferences for CVC according to training status are represented in Fig. 1b. US-guided IJV was the first choice for trained individuals (41%), in contrast to the landmark-guided subclavian approach (48.4%) for those not trained. Overall, eighty-five percent of non-trained individuals preferred landmark-guided CVC.

Physicians’ perception about strength of recommendation for US-guided IJV catheterization appears to be affected by their training status. While answers of “routine use” or “strong recommendation” were associated with trained units, “weak recommendation” was associated with non-trained units (*p* < 0.001; Fig. 1c). For the subclavian route, the same pattern was identified (*p* < 0.001).

Other invasive procedures that were reported to be “usually” US-guided included thoracentesis (49%), paracentesis (31.5%), and arterial line insertion (27%).

### Routine US-guided IJV puncture

Routine US (defined as more than two-thirds of the time) was used by 38.6% of the trained individuals compared to 16.4% of the non-trained ones (RR = 2.35 [1.31–4.23], *p* = 0.001).

Most of the factors plausibly associated with routine US-guided IJV puncture (as defined above) were statistically associated in univariate analysis (all factors except payoff). Considering multivariate analysis, two factors maintained independent association: dedicated ultrasound machine availability (RR = 2.20 [1.26–3.29], *p *=0.005) and proportion of POCUS-trained intensivists (RR = 1.91 [1.32 - 2.77], *p* = 0.001) (Additional file 2: Table S1).

### Barriers for nationwide dissemination of POCUS

One hundred thirty-three (41.3%) of the respondents answered that physicians’ training was the greatest barrier in the dissemination of ultrasonography across the Brazilian territory. When comparing the types of institutions, private hospitals responded differently (*p* = 0.012); in these institutions, limited intensivist time when considering other tasks was more relevant than medical capability or other limitations.

Main barriers to the dissemination of ultrasonography according to the training status are represented in Fig. 1d. Non-trained respondents were more likely to classify the availability of a dedicated US machine as the most relevant barrier (not training itself) (*p* = 0.021).

## Discussion

The main findings of this survey can be summarized as follows: (1) availability of a dedicated US machine in Brazilian ICUs is still suboptimal; (2) US-guided CVC insertion is the main application of POCUS, followed by echocardiography, and lung ultrasonography is rarely performed; (3) a large proportion of assessed intensivists are trained in POCUS, but as there is a predominance of courses with a short duration and workshop structure, medical residents are being trained in POCUS in an unstructured fashion); and 4) training is associated with several aspects of POCUS application, including US-guided IJV catheterization, an important marker of quality of care regarding ultrasonography in critical care medicine.

Availability of a dedicated US machine is obviously a fundamental aspect for the dissemination of POCUS. While two-thirds of the respondents in this study reported having this machine in their units, authors in other countries [8, 16] have reported higher rates. Some specific regions in Brazil and non-university hospitals were less likely to have a dedicated US machine. Our results indicated that a dedicated US machine is independently associated with adherence to US-guided IJV catheterization.

Similar rates of training in POCUS were reported from 70 to 81% in Europe and the United States [6, 8, 9], although precise information concerning the training structure and specific curriculum is scarce. Other authors [5] have reported even higher rates. Our results on this subject, however, are unique: although previous studies [5, 6, 8] have focused on specific areas within POCUS education (e.g., US-guided venous catheterization or echocardiography), we assessed general aspects of ultrasonography of the critically ill. Additionally, we aimed to directly assess the intensivists and their self-perception of training, in contrast to previous studies [9] that designed questionnaires for ICU coordinators regarding their medical staff’s capability. Medical residents in Brazil are beginning to learn ultrasonography in their education, although this is done in an unstructured and potentially heterogeneous way.

POCUS training issues are one of the major challenges of ultrasound dissemination in Brazil and other low- and middle-income countries. When heterogeneous training modalities coexist, it is difficult to ensure that all kinds of training provide comparable efficacy and competence acquisition. Standardization of training structures, both for medical residents and for those already working as intensivists, is needed, as those training structures influence intensivists’ perceptions and use of evidence-based practices.

Reaching a balance between uniform accessibility to US machines and achieving minimum requirements of quality in care delivery remains an important future objective for Brazil. Realistic simulation should be provided in a method that is scalable and replicable. Medical associations of developing countries should contextualize international recommendations on POCUS training [17, 18] for specific guidelines in Brazilian (and other developing country) contexts.

Zielezkiewicz et al. [9] demonstrated in a 1-day prevalence study in Europe that 13% of the POCUS examinations performed were for central venous puncture, and that nearly half of the CVCs were US-guided. In the present survey, we assessed physicians’ self-reporting practice, not real procedures; nevertheless, US-guided CVC insertion was identified as the major indication for POCUS by Brazilian intensivists. This behavior was also more pronounced in environments with a high level of training: the proportion of trained staff was identified as independently associated with adherence to US-guided IJV puncture. Overall adherence to routine US-guided IJV catheterization recommendations was relatively low (22.8%), compared to other critical care scenarios [19–21].

Lung ultrasonography, although infrequently reported, also showed a positive association with educational status. Lung ultrasound prevalence was negatively associated with routine chest x-ray, indicating a potential role of POCUS in reducing radiation exposure. This finding, however, is exploratory due to low adherence to lung ultrasound examination in this study. Other authors have previously reported similar data [22, 23].

Our results contrast with other studies, as US-guided CVC insertion represents the major indication for POCUS in Brazil. This could be explained by several reasons: the evidence concerning CVC guidance is solid, reducing adverse event rates and improving procedural success; the learning curve for US-guided IJV catheterization appears to be smoother than, for example, cardiac assessment and hemodynamic monitoring; some of the US machines available in Brazilian ICUs are relatively basic technology, and do not offer Doppler techniques or cardiac assessment software.

Our study has several limitations. First, inherent self-selection bias can influence our results. Physicians who already use ultrasonography were more likely to complete the survey; however, we obtained answers from a wide range of respondents from all Brazilian regions, representing private and public institutions, as well as clinical, surgical, and mixed units. Second, self-reporting, while providing direct answers from the practitioner, risks data precision. Instead of obtaining objective real-time measures, our study was based on physicians’ impressions, which could be a distorted representation of actual practices. This limitation has been previously recognized [24]. Third, as we obtained direct responses from the intensivists, large ICUs could be redundantly represented, which could also bias our results. Nevertheless, our sample consisted of intensivists working in small units (40.5% had less than 10 beds) with part time intensivist presence (47.2%), indicating that although some degree of redundancy may have occurred, it was likely of minor influence to our results. Finally, we aimed to construct direct, objective questions that could reduce misunderstanding as much as possible. Our intention was to minimize the survey burden effect [25] that could potentially minimize our return rate. Nevertheless, our questionnaire was validated in a standard way, as previously described [12].

## Conclusions

The rate of dissemination of POCUS in Brazilian ICUs is nearing rates reported in other countries. Although US-guided CVC insertion constitutes the main application of POCUS, adherence to international recommendations remains suboptimal. POCUS training, although including various relevant aspects of care, is heterogeneous, as is medical resident education regarding POCUS. Future research could address these gaps and help to better achieve POCUS dissemination in developing countries.

## Additional files

**Additional file 1.** Survey's set of questions.

**Additional file 2: Table S1.** Poisson regression model for routine* US-guided IJV catheterization.

## Abbreviations

CVC: central venous catheterization
ECOTIN: Ecografia em Terapia Intensiva (ultrasonography in intensive care)
ICU: intensive care unit
IJV: internal jugular vein
POCUS: point-of-care ultrasonography
RR: risk ratio
US: ultrasound
