# Supplementary material for: Point-of-care ultrasonography in Brazilian intensive care units: a national survey
Source: Ann Intensive Care. 2018 Apr 20;8:50. doi: 10.1186/s13613-018-0397-3 (PMC5972134; doi:10.1186/s13613-018-0397-3)
Supplement: Supplementary file 1 — Additional file 1. Survey's set of questions. [file 13613_2018_397_MOESM1_ESM.docx]

Dear colleague,

We invite you to fullfill a short questionnaire concerning point-of care ultrasound practices in your ICU.

Please, choose only ONE alternative for each question, unlesse when indicated.

Parte superior do formulário

1. Which is your institution’s profile?

University-affiliated

Public

Private

2. Which is your ICUs profile?

Mixed Clinico-Surgical

Clinical

Surgical

Trauma

Pediatrics

3. In which of the Brazil’s regions the ICU where you work is situated?

South

Southeast

Central-West

North

Northeast

4. How many ICU beds are available at your ICU?

Less than 10

11 to 20

21 to 40

More than 40

5. Among your ICU staff are there certified physicians on a daily routine basis?

Yes, full time during dayshifts

Yes, part time during dayshifts

No

6. Which was your ICU occupancy rate during tha last month?

Less than 70%

Between 70 and 80%

Between 80 and 90%

Between 90 and 95%

Above 95%

7. Do you have access to an ultrasound machine dedicated to your ICU?

No, only through demanding to other areas

Yes; only B and M mode

Yes; with spectral Doppler

Yes; with color, pulsed and continuous wave and tissue Doppler

8. Wich alternative best decribes your medical team in terms of certification in critical care?

All the physicians are intensivists certified by AMIB

Only the diarists are certified by AMIB

Only some physicians are certified (less than 3)

No physician is certified

9. How many patients do you estimate that had POCUS exams performed on a daily basis?

Less than 10%

Between 10 and 25%

Between 25 and 50%

Between 50 and 75%

More than 75%

10. Which is the most common POCUS application in your ICU?

Central venous catheterization

Lung ultrasound

Cardiac ultrasound

Abdominal ultrasouns

Nerve oprtic sheath assessment

11. In your staff, are there members with formal trainment in POCUS?

Yes, being attended ECOTIN

Yes; ECOTIN and other courses

Yes; other courses not ECOTIN

No (skip to Q14)

12. IF you answered YES to the above question, which is the trainment modality?

Short duration (1-2 days)

Between 2 days and 1 week

Between one week and one month

More than one month

13. IF you answered YES to the question 11, how many members of your staff have formal trainment in POCUS?

Less than 10%

Between 10 and 25%

Between 25 and 50%

Between 50 and 75%

More than 75%

14. Which is the incidence-density of central venous catheters (CVC) -days in your ICU?

Less than 200 CVCs-day

From 201 to 300 CVCs-day

From 301 to 500 CVCs-day

More than 501 CVCs-day

This information is not available to me

15. Which was the bloodstream infection rate in the last month?

Less than 2 / 1.000 CVC-days

Between 3 and 5 / 1.000 CVC-days

Between 6 and 10 / 1.000 CVC-days

More than 10 / 1.000 CVC-days

16. Which site is your first choice for central venous access?

Subclavian vein, landmark-based

Subclavian vein, US-guided

Internal jugular vein, landmark-based

Internal jugular vein, US-guided

Femoral vein, landmark-based

Femoral vein, US-guided

17. With which frequency internal jugular vein catheterizations are US-guided in your ICU?

Rarely, just in specific situations

Between 1/3 and 2/3

More than 2/3

Always when possible

18. With which frequency subclavian vein catheterizations are US-guided in your ICU

Rarely, just in specific situations

Between 1/3 and 2/3

More than 2/3

Always when possible

19. Among the itens below, which are available for US-guided central venous catheterization in your ICU (please mark all the options suitable)

Sterile plastic cover

Sterile gel

Customized needle guides

None of the above

20. Are there medical residents in your ICU?

Yes, including critical care

Yes, but not intensive care

No (skip to Q22)

21. IF you answered YES to the Q20, which of the below is true concerning medical residents trainment in US-guided central venous catheterization?

Learning is practice-based: there is no educational program formatted

There is a theory-based program, beyond practice at bedside

There is realistic simulation, beyond theory and practice

Only the staff are allowed to insert central catheters

22. In your opinion, which is the recommendation strength for US use for IJV catheterization?

Mandatory

Strong

Moderate

Weak

23. In your opinion, which is the recommendation strength for US use for subclavian catheterization?

 Mandatory

Strong

Moderate

Weak

24. Wich other invasive procedures are routinely US-guided in your ICU (mark all the suitable alternatives)

Arterial ine insertion

Femoral vein catheterization

Thoracentesis

Paracentesis

All of the above

25. In your ICU, the medical staff perform optic nerve sheath assessment with ultrasound?

Yes

No

26. In your ICU, the medical staff perform abdominal utrasound studies at the bedside?

Yes

No

27. How many cardiac ultrasound studies you estimate were performed last week in your ICU?

0

1-3

3-5

5-10

>10

28. How many lung ultrasound studies you estimate were performed last week in your ICU?

0

1-3

3-5

5-10

>10

29. How many patients have chest x-ray studies performed in your ICU on a routine basis?

0-25%

25-50%

50-75%

>75%

100%

30. In your ICU, POCUS exams are:

Recorded, registered in medical file and formally reported

Recorded and registered in medical file

Only registered in medical records

Not routinely registered

31. The POCUS exams performed in your ICU result in some kind of payoff?

Yes, for the institution

Yes, for the intensivist

Yes, for both the intensivist and the institution

No

32. Which of the below you believe is the greater barrier for ultrasound dissemination in ICUs across Brazil?

Medical trainment

Ultrasound machine availability

Limited intensivist time

Inexistence of payoff
