# Supplementary material for: Point-of-care ultrasonography in Brazilian intensive care units: a national survey
Source: Ann Intensive Care. 2018 Apr 20;8:50. doi: 10.1186/s13613-018-0397-3 (PMC5972134; doi:10.1186/s13613-018-0397-3)
Supplement: Supplementary file 2 — Additional file 2: Table S1. Poisson regression model for routine* US-guided IJV catheterization. [file 13613_2018_397_MOESM2_ESM.docx]

.

| **Additional file 1: Table S1. Poisson regression model for routine* US-guided IJV catheterization** | | | | | |
| --- | --- | --- | --- | --- | --- |
| **Variable** | **prevalence — n (%)** | **unadjusted RR** | **p-value** | **adjusted RR ^¶^** | **p-value** |
| US machine availability  Yes  No | 93 (46.7)  13 (11.6) | 4.03 (2.36 – 6.85)  1.00 | < 0.001 | 2.20 (1.26 – 3.29) | 0.005 |
| % of trained intensivists  > 10%  < 10% | 69 (55.2)  27 (21.8) | 2.53 (1.75 - 3.67)  1.00 | < 0.001 | 1.91 (1.32 - 2.77) | < 0.001 |

US, Ultrasound; IJV, Internal Jugular Vein; RR, risk ratios. Data are presented as risk ratios (95% confidence intervals).

**^¶^** Model adjusted for the following variables: institution's type (universitary vs. non-universitary), presence of an intensivist on a daily basis, intensivists' formal certification in critical care and payoff.

* “Routine” defined as more than two-thirds of procedures.
